# Selenium Uptake, Transport, Metabolism, Reutilization, and Biofortification in Rice

**DOI:** 10.1186/s12284-022-00572-6

**Published:** 2022-06-15

**Authors:** Lianhe Zhang, Chengcai Chu

**Affiliations:** 1 Luoyang Key Laboratory of Plant Nutrition and Environmental Ecology, Agricultural College, Henan University of Science and Technology, Luoyang, 471003 China; 2 State Key Laboratory for Conservation and Utilization of Subtropical Agro-Bioresources, College of Agriculture, South China Agricultural University, Guangzhou, 510642 China; 3 Guangdong Laboratory for Lingnan Modern Agriculture and Technology, Guangzhou, 510642 China

**Keywords:** Rice (*Oryza sativa* L.), Selenium, Se biofortification, Se metabolism, Se reutilization

## Abstract

Selenium (Se) is an essential trace element for humans and other animals. The human body mainly acquires Se from plant foods, especially cereal grains. Rice is the staple food for more than half of the world’s population. Increasing the Se concentration of rice grains can increase the average human dietary Se intake. This review summarizes recent advances in the molecular mechanisms of Se uptake, transport, subcellular distribution, retranslocation, volatilization, and Se-containing protein degradation in plants, especially rice. The strategies for improving Se concentration in rice grains by increasing Se accumulation, reducing Se volatilization, and optimizing Se form were proposed, which provide new insight into Se biofortification in rice by improving the utilization efficiency of Se.

## Background

Selenium (Se) is an essential trace element for humans and other animals (Schwarz and Foltz 1957; Rotruck et al. 1973). It was first discovered in 1817 by the Swedish chemist Jons Jakob Berzelius in the residue of sulfuric acid production. Se had been ignored chronically by scientists until its toxicity was revealed (Franke 1934). Since it was discovered that Se could prevent liver necrosis in rats, the essential physiological functions of Se in animals have been gradually revealed (Schwarz and Foltz 1957). Se exerts its antioxidant functions by forming the active site of glutathione peroxidase as selenocysteine (SeCys), thereby protecting animal tissues and cell membranes from oxidative stress damage (Rotruck et al. 1973; Rayman 2000). A low Se status in the human body reduces the immunity to many diseases and increases the susceptibility to cancers, virus infections, and heart diseases (Rayman 2000, 2012). Severe Se deficiency can even cause Keshan disease and Kashin-Beck disease (Moreno-Reyes et al. 1998; Tan et al. 2002; Oropeza-Moe et al. 2015).

Se primarily depends on selenoproteins to exert its functions in humans and animals. The human selenoproteome reveals that many selenoproteins generally participate in antioxidant and anabolic processes (Hatfield and Gladyshev 2002; Kryukov et al. 2003). The level of Se intake in Europe and some parts of China is not adequate for full expression of selenoprotein (Rayman 2007). Approximately one billion people worldwide suffer from insufficient Se intake (Combs 2001). Therefore, an adequate Se intake is crucial to prevent Se deficiency-related diseases in humans.

The human body mainly acquires Se from plant foods, especially cereal grains. Rice is the staple food for more than half of the world’s population. However, approximately 75% of rice grains provide less than 70% of the recommended daily intake of Se (Williams et al. 2009). Therefore, increasing the Se concentration in rice grains is of great importance for improving the human body’s Se intake. The Se concentration of rice grains largely depends on the Se status in the paddy soil where the rice plants are grown. The global Se concentration in soil ranges from 0.01 to 2.0 mg kg^−1^ with an average of 0.40 mg kg^−1^. However, it can be as high as 1200 mg/kg in seleniferous soils (Fiona 2007). The soil Se levels were generally divided into five grades based on the concentration range, including Se-deficient (< 0.125 mg/kg), Se-marginal (0.125–0.175 mg/kg), Se-sufficient (0.175–0.40 mg/kg), Se-rich (0.40–3.0 mg/kg), and Se-excessive (> 3.0 mg/kg) (Tan et al. 2002; Dinh et al. 2018). China has a large area of low Se soils. A saddle-shaped Se-deficient belt extends from the northeast to the southwest, and the soil Se concentration is even less than 0.13 mg/kg (Tan et al. 2002; Li et al. 2012). Therefore, it is necessary to increase the Se concentration in rice grains by applying Se in low Se areas.

Se naturally occurs as selenide, elemental Se, thioselenate, selenite, and selenate in soils (Läuchli 1993). The forms of Se are governed by various chemical and physical properties, including pH, chemical and mineralogical composition, adsorbing surfaces, and oxidation–reduction status (Neal et al. 1987). Selenate and selenite are the dominant forms of Se available to plants in soils. The availability of selenite in the soil is usually many times lower than that of selenate due to being adsorbed by organic matter and Fe hydrated oxides (Coppin et al. 2006; Keskinen et al. 2013). In well-aerated alkaline soils, selenate is the dominant Se form. In neutral and acid soils, selenite is the dominant Se form (Neal et al. 1987; Mikkelsen et al. 1989). Under reducing conditions, most of the selenate is readily converted to selenite (Elrashidi et al. 1987). Therefore, rice plants mainly take up selenite under flooded conditions in paddy fields. After selenite is taken up by plants, it can be converted into organic Se such as selenomethionine (SeMet) and unspecifically involved in protein synthesis (Terry et al. 2000; Zhang et al. 2019). Se-containing proteins are degraded by different types of proteases during leaf senescence to release SeMet. SeMet is an analog of methionine (Met) and has a common transporter with Met (Gits and Grenson 1967). Met has been demonstrated to be transported by amino acid transporters (Taylor et al. 2015). Therefore, SeMet can also be transported into grains by amino acid transporters. In addition, Se can be further converted into dimethyl selenides (DMSe) and volatilized, resulting in a decrease in the accumulation of Se in rice grains (Zayed et al. 1998). Se accumulation in rice grains involves a series of complex processes, including uptake, transport, subcellular distribution, and retranslocation. It requires fine cooperation of multiple transporters specifically localized to  the cell membranes or subcellular membranes of different organs and tissues. In this review, we summarize the research progresses on the mechanisms of Se uptake, transport, subcellular distribution, retranslocation, volatilization, and Se-containing protein degradation in plants, especially rice, and propose the strategies for improving Se accumulation in rice grains, which is crucial for promoting Se biofortification in rice.

## The Se Uptake and Transport in Plant

### Selenate Uptake and Transport

**Fig. 1:**
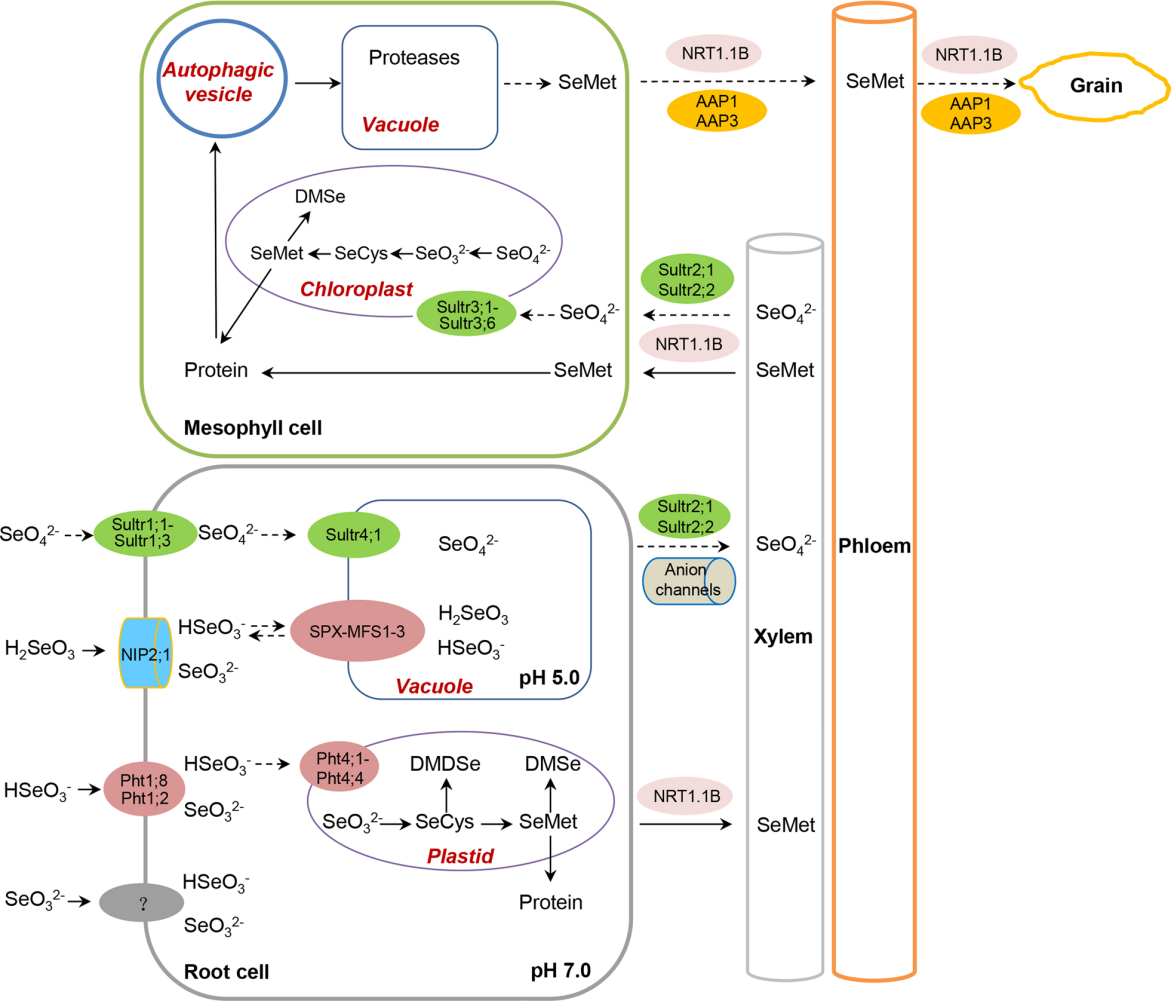
The uptake, transport, subcellular distribution, retranslocation, and volatilization of Se in rice. Selenate is taken up through Sultr1;1, Sultr1;2, and Sultr1;3, located in the root epidermal cell membrane, a small part of selenate enters the vacuole through Sultr4;1 located in the tonoplast, and most of it is transported to the leaves through Sultr2;1 and Sultr2;2 located in the parenchyma cell membrane of the xylem, finally enters the chloroplast through Sultr3;1, Sultr3;2, Sultr3;3, Sultr3;4, Sultr3;5, and Sultr3;6 located in the chloroplast membrane, where they are converted into SeCys and SeMet to participate in protein synthesis in a non-specific manner, and can also be further converted into DMSe and volatilized. Rice roots can take up HSeO_3_^−^ and H_2_SeO_3_ through OsPht1;2 (OsPT2) and OsNIP2;1, respectively. After selenite enters the cytoplasm, it mainly exists in the form of HSeO_3_^−^ and SeO_3_^2−^; part of the selenite enters the vacuole through OsSPX-MFS1/3 and OsVPE1/OsVPE2 located in the tonoplast. Selenite mainly exists in the form of H_2_SeO_3_ and HSeO_3_^−^ in the vacuole; most of it is transported to the plastid by OsPHT4;1-OsPHT4;4 and OsPHT2;1 and converted into SeCys and SeMet, and then participate in protein synthesis non-specifically, and can also be further converted into DMSe and volatilizes. Part of SeMet is transported to shoots through NRT1.1B and participates in protein synthesis. In senescent leaves, protein is encapsulated in autophagic vesicles and transported to vacuoles, degraded into SeMet by proteases, and transported to rice grains through OsAAP1, OsAAP3, and OsNRT1.1B. Sulfate transporters,  ; OsNIP2;1,  ; Phosphate transporters,  ; NRT1.1B,  ; Amino acid transporters,  ; Anion channels,  Solid lines correspond to identified transporters and dashed lines correspond to potential transporters

Earlier physiological studies revealed that selenate uptake was an active process because respiratory inhibitors and low temperature almost completely inhibited selenate uptake (Ulrich and Shrift 1968; Shrift and Ulrich 1969). Sulfate can largely inhibit selenate uptake, suggesting that selenate shares a common uptake mechanism with sulfate, with both being taken up by proton gradient-driven sulfate transporters (Terry et al. 2000; Hawkesford 2003; Sors et al. 2005). The sulfate transporter gene family could be classified into four distinguishable groups according to phylogenetic analysis of the plant gene or amino acid sequences (Hawkesford 2003; Buchner et al. 2004). Group 1 sulfate transporters are high-affinity transporters responsible for sulfate uptake, and the genes of these transporters are mainly expressed in root tissues and induced by sulfur deficiency (Smith et al. 1995, 1997; Takahashi et al. 2000; Howarth et al. 2003). In Arabidopsis, the high-affinity sulfate transporters Sultr1.1 and Sultr1.2 locate in the root hairs, epidermis, and cortical cell layers. They are responsible for sulfate uptake from soil under sulfur-deficient conditions (Takahashi et al. 2000; Hawkesford 2003; Buchner et al. 2004) (Table 1). Sultr1;2 was identified by screening selenate-resistant mutants, which mediated selenate uptake (Shibagaki et al. 2002; El Kassis et al. 2007). In rice, the high-affinity sulfate transporter genes *OsSultr1;1*, *OsSultr1;2*, and *OsSultr1;3* were identified, and their expressions in roots are regulated by sulfate status (Buchner et al. 2004; Kumar et al. 2011; Réthoré et al. 2020) (Table 1). OsSultr1;1 was demonstrated to transport sulfate by expressing in heterologous systems yeast and Arabidopsis (Kumar et al. 2019). Due to the highly similar chemical properties between sulfate and selenate, the uptake of selenate by rice roots is most likely through OsSultr1;1, OsSultr1;2, and OsSultr1;3 (Fig. 1, Table 1). However, sulfate transporters are selective in taking up sulfate and selenate (Ferrari and Renosto 1972).

**Table 1:** Identified and potential transporters and channels for Se uptake, transport, and subcellular distribution

Functions	Type of transporters	Tissue or subcellular localization
Selenate uptake (group 1 sulfate transporters)	*AtSultr1;1*	Root hairs, epidermis, and cortical cell layers (Takahashi et al. 2000)
	Sultrl;2	Root cortex, root tip and lateral roots (Shibagaki et al. 2002; Yoshimoto et al. 2002)
	AtSultr1;3^*^	Sieve element-companion cell complexes of the phloem in cotyledons and roots (Yoshimoto et al. 2003)
	*OsSultr1;1-OsSultr1;3*	Root (Kumar et al. 2011; Réthoré et al. 2020)
Selenate transport (group 2 sulfate transporters)	*AtSultr2;1*	Xylem parenchyma cells of leaves and roots (Takahashi et al. 2000)
	*AtSultr2;2*	Root phloem and leaf vascular bundle sheath cells (Takahashi et al. 2000)
	*OsSultr2;1-OsSultr2;2*	Vascular tissues (Dixit et al. 2015)
Selenate transport (channels)	*Anion channels*	Xylem parenchyma cells (Gilliham and Tester 2005)
Selenate subcellular distribution (group 3 sulfate transporters)	*AtSultr3;1-AtSultr3;5*	Chloroplast (Hawkesford 2003; Buchner et al. 2004; Cao et al. 2013)
	*OsSultr3;1-OsSultr3;6*	
Selenate subcellular distribution (group 4 sulfate transporters)	*AtSultr4;1-AtSultr4;2*	Tonoplast (Hawkesford 2003; Buchner et al. 2004)
	*OsSultr4;1*	
Uptake (group 1 phosphate transporters)	OsPht1;2	Root epidermal cells and steles in primary and lateral roots (Zhang et al. 2014)
	OsPht1;8	Root tips, lateral roots, leaves, stamens, caryopses (Jia et al. 2011)
Uptake (NIP subfamily)	OsNIP2;1	Plasma membrane of the distal side of both exodermis and endodermis cells (Ma et al. 2006)
SeMet transport (PTR family)	OsNRT1.1B	Vascular tissues of roots, leaf sheaths, leaf blades and culms (Hu et al. 2015)
Subcellular distribution (group 4 phosphate transporters)	*AtPht4;1-AtPht4;5*	Plastid envelope (Guo et al. 2008)
	*OsPht4;1-OsPht4;4*	Inner chloroplast membrane (Li et al. 2020)
Subcellular distribution (group 2 phosphate transporters)	*OsPht2;1*	Chloroplast (Liu et al. 2020)
Subcellular distribution (group 5 phosphate transporters or SPX-MFS proteins)	*AtSPX-MFS 1–3*	Tonoplast (Liu et al. 2020)
	*OsSPX-MFS 1–3*	Tonoplast (Wang et al. 2012, 2015; Xu et al. 2019)

Italic fonts represent potential transporters and channels. ^*^Represents that AtSultr1;3 is a potential transporter responsible for selenate transport

Similar to sulfate, selenate is taken up by root epidermal cells and transported radially to the stele via the apoplastic and symplasmic pathways (Takahashi et al. 2000). Apoplastic transport to the stele is restricted by Casparian bands in the endodermal cell wall, limiting the uptake of ions from the apoplast by endodermal cells (White 2001; Moore et al. 2002). The symplasmic pathway plays a key role in delivering most ions to the xylem, with radial transport of ions to adjacent root cells via the plasmodesmata (Lucas and Wolf 1993). Likewise, selenate is also transported in the symplast via plasmodesmata. After radial transport of selenate to the stele via the symplasm, selenate is released into xylem vessels across the plasma membrane of xylem parenchymal cells.

Group 2 sulfate transporters, including Sultr 2;1 and Sultr 2;2, are mainly located in the vascular tissues and are responsible for sulfate transport (Takahashi et al. 2000). Sultr 2;1, located in the xylem parenchyma cells of leaves and roots, participates in sulfate transport from roots to shoots via the xylem (Takahashi et al. 2000). Therefore, selenate may be loaded into the stele via Sultr 2;1 (Fig. 1, Table 1). Furthermore, since anion channels can facilitate the movement of sulfate from the cytoplasm of the xylem parenchyma cells to the xylem vessels along the electrochemical gradient, selenate may also be loaded into the xylem in a similar manner (Gilliham and Tester 2005) (Fig. 1, Table 1). Sultr2;1 is colocalized with Sultr 3;5 in xylem parenchyma and pericycle cells in roots. Co-expression of *Sultr2;1* and *Sultr3;5* provides a maximum capacity of sulfate transport, which facilitates retrieval of apoplastic sulfate to the xylem parenchyma cells in the vasculature of roots and may contribute to the root-to-shoot transport of sulfate (Kataoka et al. 2004a). Sulfate released from the xylem is retrieved by Sultr 2;1 to xylem parenchyma cells and vascular bundle sheath cells and enters mesophyll cells through plasmodesmata (Kataoka et al. 2004a). Sultr2;2, expressed in the root phloem and leaf vascular bundle sheath cells, participates in the transport of sulfate released from xylem vessels into the mesophyll cells (Takahashi et al. 2000). Therefore, Sultr 2;1 and Sultr 2;2 may also retrieve selenate from xylem vessels and transport it into mesophyll cells (Fig. 1). Group 2 sulfate transporters in rice include OsSultr 2;1 and OsSultr 2;2. The selenate taken up by the roots is most likely to be transported from roots to shoots by these transporters, which remains to be experimentally confirmed (Fig. 1, Table 1).

## Selenite Uptake and Transport

Earlier physiological studies revealed that respiratory inhibitors and low temperature could largely inhibit selenite uptake at pH 4.0, suggesting that selenite uptake was an active process (Ulrich and Shrift 1968). However, later studies indicated that the respiratory inhibitors and low temperature could only slightly inhibit selenite uptake, indicating that selenite uptake was a passive process (Arvy 1993). Therefore, previous studies on the physiological mechanism of selenite uptake by plants were inconsistent. The conflicting results of previous studies should be attributed to the neglect of the existence of different Se forms in selenite solutions, which vary with pH. At low selenite concentrations, H_2_SeO_3_, SeO_3_^2−^, and HSeO_3_^−^ coexist in aqueous solutions and can also be converted into each other with pH to maintain ion balance (Zhang et al. 2006a). According to the ionization constant of H_2_SeO_3_ (K1 = 2.7 × 10^–3^, K2 = 2.5 × 10^–7^), the proportion of different Se species in different pH media can be calculated. For example, H_2_SeO_3_ and HSeO_3_^−^ constitute about 27% and 73% at pH 3.0; HSeO_3_^−^, SeO_3_^2−^, and H_2_SeO_3_ constituted about 97.2%, 2.4%, and 0.04% at pH 5.0; SeO_3_^2−^ and HSeO_3_^−^ constitute about 96.2% and 3.8% at pH 8.0. The H_2_SeO_3_, HSeO_3_^−^, or SeO_3_^2−^ species represented the dominant forms of selenite in the solution at different pH, respectively (Zhang et al. 2006a, 2010b). Respiratory inhibitors and low temperature largely inhibited selenite uptake at pH 5.0, suggesting that selenite (HSeO_3_^−^) uptake at pH 5.0 was coupled with energy metabolism. Furthermore, the excellent fit of selenite (HSeO_3_^−^) uptake data to a Michaelis–Menten equation suggested that selenite was taken up by a transporter-mediated process involving selective membrane binding sites (Zhang et al. 2006a, 2010b). Selenite uptake was inhibited by phosphate in hydroponic experiments (Hopper and Parker 1999). Physiological experiments further revealed that selenite uptake could be regulated by phosphate transporters (Li et al. 2008). OsPHT1;2 (OsPT2) is a phosphate transporter responsible for Pi transport in rice (Ai et al. 2009). OsPT2 is localized in root epidermal cells and steles in primary and lateral roots (Ai et al. 2009; Zhang et al. 2014). Interestingly, *OsPT2*-overexpressing and knock-down plants displayed significantly increased and decreased rates of selenite uptake, demonstrating that OsPT2 is involved in selenite uptake (Zhang et al. 2014). OsPHT1;8 (OsPT8) is a high-affinity Pi transporter, expressed in various tissues and organs, including root tips, lateral roots, leaves, stamens, caryopsis, and germinated seeds, which is involved in Pi homeostasis in rice (Jia et al. 2011). Overexpression of *OsPT8* increased Se concentration in tobacco plants, suggesting that OsPT8 is also involved in selenite uptake and transport (Song et al. 2017) (Fig. 1, Table 1).

 Respiratory inhibitors and low temperature had little effect on selenite uptake at pH 3.0 and pH 8.0, suggesting that selenite uptake was an energy-independent process at these pHs (Zhang et al. 2010b). The amount of selenite taken up increased linearly in proportion to the increasing Se concentration in the absorption solution, suggesting that selenite uptake was possibly a passive process at pH 3.0 or pH 8.0 (Zhang et al. 2006a, 2010b). The result was partly consistent with previous results (Arvy 1993). HgCl_2_ and AgNO_3_ are well-known aquaporin blockers, which could inhibit the uptake of selenite largely at pH 3.0 in rice and maize (Niemietz and Tyerman 2002; Zhang et al. 2006a, 2010b). The inhibition of selenite uptake by HgCl_2_ or AgNO_3_ might be related to the inhibition of aquaporin activity, suggesting that H_2_SeO_3_ is taken up through aquaporin (Zhang et al. 2006a, 2010b). Furthermore, a silicon influx transporter OsNIP2;1 (Lsi1) identified in rice belongs to the nodulin 26-like intrinsic membrane protein (NIP) subfamily of aquaporins (Ma et al. 2006). OsNIP2;1 is localized to the plasma membrane of the distal side of both exodermis and endodermis cells and is constitutively expressed in roots. Expression of *OsNIP2;1* in yeast enhanced selenite uptake at pH 3.5 and 5.5, but not at pH 7.5. Defect of Si efflux transporter OsNIP2;2 did not affect selenite uptake, demonstrating that Si influx transporter OsNIP2;1 is permeable to selenite (Zhao et al. 2010) (Fig. 1, Table 1).

Previous studies indicated that after selenite was taken up by plant roots, only a small amount of Se was transported to the shoots, and most of the Se remained in the roots (Arvy 1993; Zayed et al. 1998; Li et al. 2008). When rice seedlings were supplied with selenite, large amounts of SeMet were detected in the roots, only a small amount of MeSeCys was found, suggesting that selenite is mainly converted to SeMet in plastids (Zhang et al. 2019). In addition, SeMet is mainly detected in leaves and sheaths, indicating that SeMet is the dominant form of transport when supplied with selenite (Zhang et al. 2019). OsPT2 and OsPT6 are both highly expressed in stele cells of rice roots and play essential roles in Pi root-to-shoot translocation and Pi homeostasis in the plant (Ai et al. 2009). Although more selenite was present in roots, no selenite was detected in leaf blades and leaf sheaths, indicating that selenite was not transported to shoots by OsPT2 and OsPT6 (Zhang et al. 2019). Selenite is also not transported into the shoots of pakchoi after being taken up by the roots (Yu et al. 2019). The reason why selenite cannot be transported to shoots should be that selenite has been converted to organic Se before being transported (Yu et al. 2019; Zhang et al. 2019).

## Organic Se Uptake and Transport in Plant

Organic Se is also one of the forms of Se available to plants (Abrams and Burau 1989). Organic Se accounts for about 40% of the total Se in the soil and 50% of the soluble Se extracted from the soil. It is stable in the soil and does not change appreciably with the variation of soil conditions (Yamada et al. 1998). Organic Se may be derived from decomposing plant tissues or incorporating into the organic fraction from inorganic Se abiotically or microbiological activity (Abrams and Burau 1989). SeMet is the dominant organic Se identified in soils (Abrams and Burau 1989; Abrams et al. 1990a). SeMet uptake by wheat seedlings followed Michaelis–Menten kinetics and was coupled to metabolism evidenced by inhibition of metabolic inhibitors and by anaerobic conditions (Abrams et al. 1990b). NRT1.1B, a member of the PTR family, encodes a protein containing a peptide-transporter domain. *NRT1.1B* is predominantly expressed in the vascular tissues of rice roots, leaf sheaths, leaves, and culms. Together with its plasma membrane localization, NRT1.1B was demonstrated to be involved in root-to-shoot nitrate transport (Hu et al. 2015). Furthermore, NRT1.1B has SeMet transport activity and also mediates the root-to-shoot translocation of SeMet in rice (Fig. 1, Table 1). *NRT1.1B* overexpression significantly improved not only the Se concentrations in shoots but also in grains (Zhang et al. 2019).

## Subcellular Distribution of Different Forms of Se

Plastids, especially chloroplasts, are the main site of reductive assimilation of sulfur and Se in plants (Terry et al. 2000). Group 3 sulfate transporters in Arabidopsis, including Sultr3;1, Sultr3;2, Sultr3;3, Sultr3;4, and Sultr3;5, are localized in the chloroplasts responsible for sulfate transport into chloroplasts (Cao et al. 2013; Chen et al. 2019) (Table 1). Single knockout mutants of group 3 sulfate transporters showed reduced chloroplast sulfate uptake, suggesting that these sulfate transporters may also be involved in chloroplastic sulfate transport, the contribution of sulfate influx into chloroplasts by Sultr3;2, Sultr3;3, and Sultr3;4 was estimated at 74%, 66%, and 69% of the wild type, respectively (Cao et al. 2013). Sulfate uptake by chloroplasts of the quintuple mutant was reduced by more than 50% compared with the wild type (Chen et al. 2019). However, sulfate uptake was hardly detectable with *Sultr3;5* expression alone, whereas cells coexpressing both *Sultr3;5* and *Sultr2;1* exhibited substantial uptake activity that was considerably higher than with *Sultr2;1* expression alone (Kataoka et al. 2004a). Due to the highly similar properties of selenate and sulfate, selenate in the chloroplasts is most likely transported from the cytoplasm by group 3 sulfate transporters. In rice, group 3 sulfate transporters include OsSultr3;1, OsSultr3;2, OsSultr3;3, OsSultr3;4, OsSultr3;5, and OsSultr3;6 (Buchner et al. 2004) (Table 1). Overexpression of *OsSultr3;3* in yeast and *Xenopus* oocytes revealed that OsSultr3;3 had no sulfate transporter activity. However, disruption of OsSultr3;3 reduces sulfate and cysteine concentrations, whereas no significant differences in total S concentration were observed (Zhao et al. 2016). Although these transporters have not been demonstrated to transport selenate, cytosolic selenate is most likely transported by them into the chloroplasts (Fig. 1).

The efflux of sulfate from the vacuoles maintains cytosolic sulfate homeostasis and promotes transport toward the xylem vessels. In group 4, there are two sulfate transporters Sultr4;1 and Sultr4;2 in Arabidopsis but only one OsSultr4;1 in rice (Buchner et al. 2004) (Table 1). Sultr4;1 and Sultr4;2 are tonoplast-localizing transporters in Arabidopsis (Kataoka et al. 2004b). Sultr4;1 is the main transporter facilitating the unloading of vacuolar sulfate reserve in the roots, and Sultr4;2 may play similar and supplementary roles in supporting the Sultr4;1 function at the tonoplast. The contribution of Sultr4;2 was estimated 15% of the Sultr4;1 function (Kataoka et al. 2004b). OsSultr4;1 may be located in the tonoplast of rice and is responsible for the transport of selenate to the vacuole, but further confirmation is needed (Fig. 1, Table 1).

After selenite is taken up by the roots, it is readily converted to SeMet catalyzed by sulfur-metabolizing enzymes (Terry et al. 2000). Since GSH reductase, Cys synthase, cystathionine-γ-synthase, and cystathionine-β-lyase are primarily present in plastids, suggesting that selenite enters plastids soon after being taken up by the root (Takahashi and Saito 1996; Terry et al. 2000). When rice seedlings were supplied with selenite for 3 d, large amounts of selenite were still detected in the roots (Zhang et al. 2019). Since GSH, O-acetylserine, and NADPH are present in the cytoplasm (Foyer et al. 2001; Chai et al. 2006; Hider and Kong 2011; Li et al. 2022), so selenite is readily reduced to selenol (GS-SeH) by GSH and NADPH (Terry et al. 2000). Therefore, a large amount of selenite is unlikely to exist in the cytoplasm for a long time but in the vacuole.

The distribution of selenite in organelles largely depends on the pH in the cytosol. At neutral pH in the cytosol, there are mainly two forms of Se, SeO_3_^2−^ and HSeO_3_^−^, which account for 71.4% and 28.6%, respectively (Zhang et al. 2006a, 2010b) (Fig. 1). Previous studies indicated that SeO_3_^2−^ could enter the root passively at a slow speed (Zhang et al. 2006a, 2010b). Likewise, SeO_3_^2−^ also slowly enters the vacuole and plastid. Under pH-neutral conditions, SeO_3_^2−^ and HSeO_3_^−^ coexist in a specific ratio in the selenite solution. The two chemical forms of Se can be converted into each other to maintain ion balance. Since the uptake rate of HSeO_3_^−^ is much higher than that of SeO_3_^2−^, the ion balance between SeO_3_^2−^ and HSeO_3_^−^ is broken, resulting in more SeO_3_^2−^ being converted to HSeO_3_^−^. Therefore, selenite in the cytosol is mainly transported in the form of HSeO_3_^−^ into organelles such as vacuoles and plastids.

Previous studies have demonstrated that Pi transporters have transport activities for selenite (Li et al. 2008; Zhang et al. 2014; Song et al. 2017). The Arabidopsis PHT4 proteins mediate Pi transport in yeast with high specificity. PHT4;1-PHT4;5 localize to the plastid envelope and regulates Pi entry into the plastid (Guo et al. 2008). Rice OsPHT4;1-OsPHT4;4 localize to the inner chloroplast membrane and are involved in the distribution of Pi between the cytoplasm and chloroplast (Li et al. 2020) (Table 1). In addition, the low-affinity Pi transporter OsPHT2;1 functions as a chloroplast-localized low-affinity Pi transporter, mediating Pi entry into the chloroplast (Liu et al. 2020). The Arabidopsis SPX-MFS protein, designated as PHOSPHATE TRANSPORTER 5 family (PHT5), also named Vacuolar Phosphate Transporter (VPT), functions as vacuolar Pi transporters (Liu et al. 2015, 2016). Rice SPX-MFS family contains four genes, including *OsSPX-MFS1*, *OsSPX-MFS2*, *SPX-MFS3*, and *SPX-MFS4* (Secco et al. 2012). OsSPX-MFS1, OsSPX-MFS1, and SPX-MFS3 localize to the tonoplast and are responsible for vacuolar Pi influx or efflux (Wang et al. 2012, 2015; Xu et al. 2019) (Fig. 1, Table 1). Therefore, the selenite in the form of HSeO_3_^−^ in the cytosol may enter the vacuole and the plastid through these Pi transporters located in the tonoplast and chloroplast membrane (Fig. 1, Table 1).

## The Release of SeMet from Protein Degradation

The leaves mainly accumulated SeMet when rice seedlings were supplied with selenite (Zhang et al. 2019). A large amount of SeMet non-specifically replaces Met to participate in protein synthesis in the leaves. During leaf senescence, Se-containing proteins are degraded by proteases, and SeMet is released and retranslocated to the developing grains. Se concentration in brown rice was positively correlated with the total Se in shoots (Zhang et al. [Bibr CR140][Bibr CR141]). Therefore, the higher the Se concentration in leaf blades, the more SeMet released by protein degradation during leaf senescence, the more SeMet transported to the grain, and the higher the grain Se concentration. Previous studies have revealed that most of the nitrogen in the grains mainly derives from the reutilization of nitrogen in shoots (Palta and Fillery 1995; Kichey et al. 2007; Gregersen et al. 2008). Protein degradation in leaves is a prerequisite for nitrogen reutilization (Gregersen et al. 2008). Up to 75% of the proteins in the leaves are in the chloroplast, including thylakoid membrane proteins and matrix proteins, most of which are ribulose-1,5-bisphosphate carboxylase (RuBisCO, EC 4.1.1.39). The nitrogen released from senescent leaves mainly comes from the degradation of RuBisCO and other proteins in chloroplasts (Otegui 2018). Improving the reutilization capacity of RuBisCO can enhance the efficiency of nitrogen utilization (Desclos et al. 2009; Girondé et al. 2015). Since Se and nitrogen coexist in proteins in the form of SeMet and various amino acids, respectively, protein degradation in senescent leaves may simultaneously affect the reutilization of Se and nitrogen. Regulating the degradation of proteins, especially chloroplast proteins, may also improve the reutilization efficiency of Se.

Protein degradation in senescent leaves is closely associated with protease activities (Roberts et al. 2012; Diaz-Mendoza et al. 2016). During the senescence of leaves, a large number of proteases, including cysteine proteases, serine proteases, aspartic proteases, metalloproteases, and threonine proteases, are induced (Guo et al. 2004; Roberts et al. 2012). At least one or more proteases are distributed in the cytoplasm, nucleus, chloroplast, mitochondria, endoplasmic reticulum, Golgi apparatus, and cell wall (Diaz-Mendoza et al. 2016). The vacuole is the main compartment where proteins are hydrolyzed into amino acids (Masclaux-Daubresse et al. 2017). Autophagy is considered to be an important mechanism for the selective degradation of chloroplasts in the vacuole (Xie et al. 2015; Otegui 2018; Zhuang and Jiang 2019). Chloroplasts are encapsulated in different vesicles by autophagy and then are transported to vacuoles for complete degradation (Fig. 1). Cysteine proteases and serine proteases are the main proteases in vacuoles. During plant senescence, the expressions of cysteine protease genes are greatly increased (Bhalerao et al. 2003; Guo et al. 2004; Diaz-Mendoza et al. 2016). Under continuous dark conditions, at least four vacuolar cysteine protease genes in senescent wheat leaves were induced (Martínez et al. 2007). In Arabidopsis and soybean leaves, cysteine protease activity is high in senescence-associated vacuoles (Otegui et al. 2005; Martínez et al. 2007). The cysteine protease SAG12 is highly expressed in natural aging tissues and exists in senescence-associated vacuoles (Guo et al. 2004; Otegui et al. 2005; Parrott et al. 2007). Cysteine protease in senescent wheat leaves could degrade the large subunit of RuBisCO into 50 kDa fragments (Thoenen et al. 2007). Serine proteases are the most abundant proteases in plants (Van der Hoorn 2008; Roberts et al. 2012). During the senescence of wheat leaves, serine protease genes are induced and play a vital role in the reutilization of nitrogen (Chauhan et al. 2009; Roberts et al. 2011). In barley senescent leaves induced by girding, the expression of serine protease is induced to accelerate leaf senescence and promote the degradation of RuBisCO and membrane proteins (Parrott et al. 2007). In senescent leaves, a large number of protease genes are induced to express, and the enzyme activities are increased, thereby accelerating protein degradation. Increasing the activity of serine protease and cysteine protease can increase the efficiency of nitrogen reutilization (Poret et al. 2019). Therefore, mining the proteases related to Se reutilization, especially serine proteases and cysteine proteases, is expected to promote protein degradation by regulating their gene expression to release more SeMet, thereby increasing grain Se concentrations.

## Retranslocation of SeMet from Senescent Leaves to Grains

Plant leaf senescence and nutrient reutilization are closely related processes. Senescence is also a process of nutrient retranslocation from leaves to reproductive organs. The protein degradation products in senescent leaves are primarily transported into the grain in the form of amino acids and small peptides (Roberts et al. 2012). Since leaf Se is predominantly present in protein as SeMet, like other amino acids, SeMet is released from protein degradation in senescent leaves before it can be transported to developing grains. The expression of multiple amino acid and peptide transporter (AAP and PTR subfamily transporters) genes was up-regulated in senescent leaves of Arabidopsis (Marmagne et al. 2016). AtAAP1 is responsible for the uptake of amino acids by the embryo in Arabidopsis and is important for storage protein synthesis and seed yield (Sanders et al. 2009). Among the AAP subfamily members whose expression was up-regulated, AtAAP2 was involved in the reutilization of nitrogen in senescent leaves, and grain nitrogen concentration was reduced in *aap2* mutants (Zhang et al. 2010a). *AtAAP8* is expressed in the phloem of leaves and is located to the plasma membrane of mesophyll cells. In the *aap8* mutants, the amino acid concentrations in phloem and grains were decreased, indicating that AtAAP8 plays an important role in the source-to-sink partitioning of nitrogen (Santiago and Tegeder 2016). In rice, OsAAP1 is localized to the plasma membrane and nuclear membrane and is highly expressed in roots, axillary buds, leaves, and panicles. Overexpression of *OsAAP1* may promote the transport of neutral amino acids from straw to grains (Ji et al. 2020). *OsAAP3* was mainly expressed in roots, leaves, leaf sheaths, culms, and panicles (Lu et al. 2018). Elevated expression of *OsAAP3* leads to amino acid accumulation in rice grains (Lu et al. 2018). *OsAAP6* is expressed in the vascular bundles in the flag leaves of rice at the heading stage. OsAAP6 can increase the concentration of grain storage protein and total amino acids, suggesting that OsAAP6 is involved in the transport of amino acids from leaves to grains (Peng et al. 2014). Improved amino acid transport from senescent leaves to grains can increase the grain protein concentration (Kade et al. 2005). SeMet, an analog of Met, shares a common transporter with Met (Gits and Grenson 1967). OsAAP1, OsAAP3, OsAAP7, and OsAAP16 have transport activity for Met (Taylor et al. 2015). Therefore, it is very likely that OsAAP1 and OsAAP3 are involved in the transport of SeMet from senescent leaves to grains. In addition, the peptide transporter NRT1.1B is expressed in the vascular tissues of leaf sheaths, leaves and culms, and is not only involved in the transport of SeMet from roots to shoots, but may also be involved in the transport of SeMet from leaves to grains and increases the Se concentration in grains (Zhang et al. 2019) (Fig. 1).

## Plant Se Volatilization

### Plant Se Volatilization is Ubiquitous

Se volatilization in plants is a ubiquitous phenomenon during growth, harvest, drying, and storage stages. The loss of Se was as high as 66% during the air-drying process after the plants were collected, and it varied with seasons and growth stages (Beath et al. 1937). Plant volatile Se was mainly released through leaves, and the volatilization rate varied throughout the day (Lewis et al. 1966). 0.5–3.0% of the total Se in the shoots and roots supplied with selenate and selenite were released in volatile form when dried at 70 °C for 48 h (Evans and Johnson 1967), while dry heating of cereal grains led to 7–23% losses of Se (Higgs et al. 1972). Se losses reached 4–73% when the grains of barley, corn, and wheat were stored for 3 to 5 years (Moxon and Rhian 1938). The release of volatile Se compounds has been demonstrated during plant growth and development (Lewis et al. 1966; Evans et al. 1968; Zayed et al. 1998; de Souza et al. 2000). Se volatilization is greatly affected by soil Se concentrations, plant species, growth stage, organs, physiological state, and Se-supplied forms (Beath et al. 1937; Zayed et al. 1998; de Souza et al. 2002). Se hyperaccumulators growing in seleniferous soils can accumulate and volatilize large amounts of Se (Beath et al. 1937). Se non-hyperaccumulators could also volatilize and release Se, which varies with plant species and Se forms. The effect of Se forms on Se volatilization rate may be attributed to be in different stages of Se metabolism (Zayed et al. 1998).

## Plant Volatile Se Forms

**Fig. 2:**
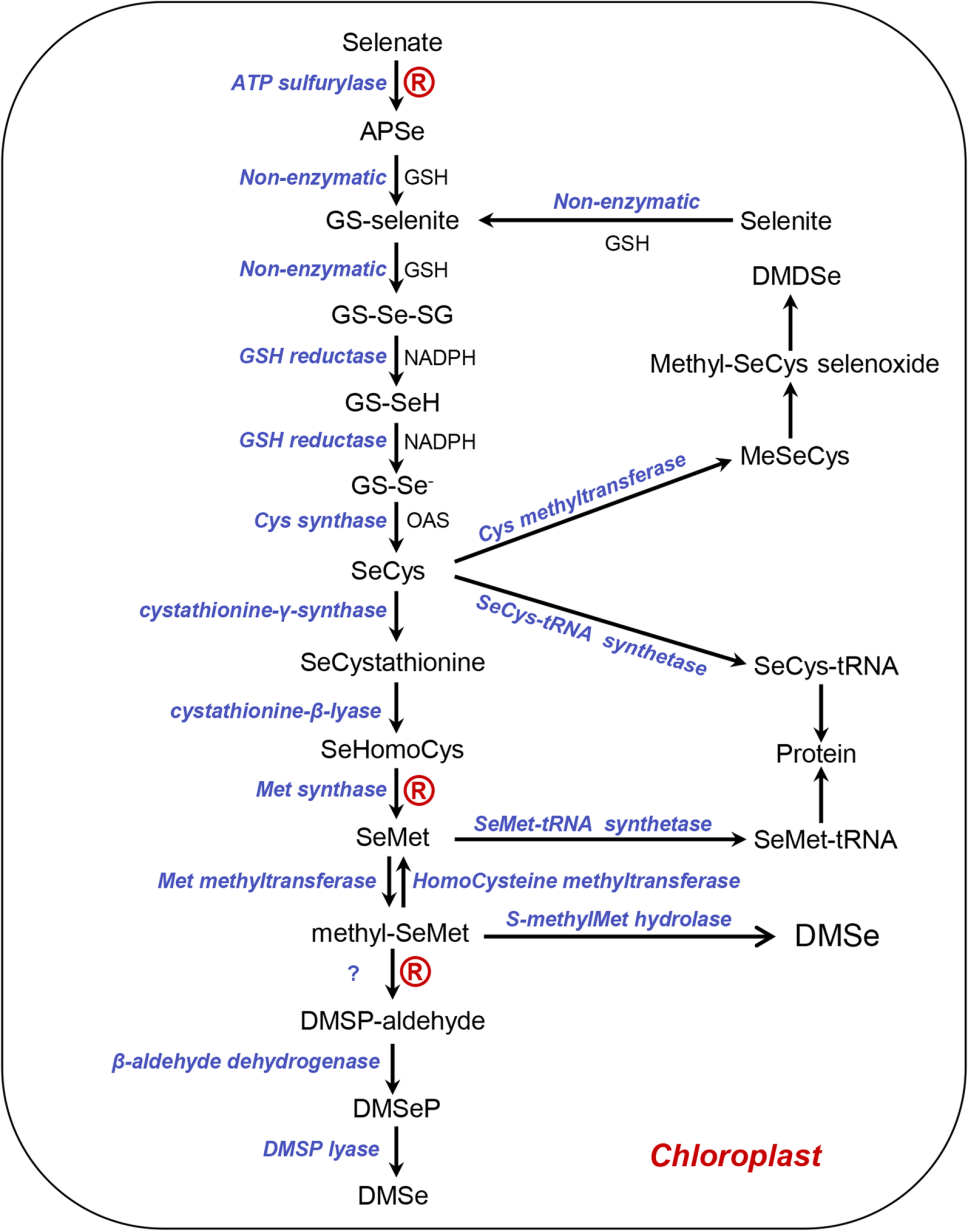
The metabolism of selenate and selenite in chloroplasts or plastids (Terry et al. 2000). Selenate is first reduced to adenosine 5-phosphoselenate (APSe) by ATP sulfurylase (EC: 2.7.7.4) and then further reduced nonenzymatically to GSH-conjugated selenite (GS-selenite). Selenite is also reduced nonenzymatically to GS-selenite. The GS-selenite is reduced to selenodiglutathione (GS-Se-SG) by GSH, and GS-Se-SG is further reduced to selenol (GS-SeH) by NADPH and subsequently to GSH-conjugated selenide (GS-Se^−^) by GSH reductase. SeCys is synthesized from GS-Se^−^ and O-acetylserine catalyzed by Cys synthase. SeMet may be synthesized from SeCys via SeCystathionine and SeHomoCys catalyzed by cystathionine-γ-synthase, cystathionine-β-lyase, and Met synthase. SeCys is methylated to methyl-SeMet by Cys methyltransferase. SeMet is methylated to methyl-SeMet, and is further converted into dimethylselenonium propionate (DMSeP) by DMSeP lyase, and then cleaved to DMSe by S-methylMet hydrolase and volatilized. R represents the rate-limiting step

After selenate or selenite is converted to SeCys in the chloroplast, it can be further converted to MeSeCys and MeSeMet, and finally to volatile Se compounds such as DMSe and dimethyldiselenide (DMDSe) (Terry et al. 2000; Kubachka et al. 2007). The volatile Se compounds are mainly DMSe, which is released directly from the intermediate metabolites of SeMet, namely MeSeMet and dimethylselenoniopropionate (DMSeP) (Terry et al. 2000). The DMSe arises from MeSeMet cleaved by S-methyl-Met hydrolase, suggesting that MeSeMet is a precursor of DMSe (Lewis et al. 1974). DMSeP is an analog of dimethylsulfoniopropionate (DMSP). MeSeMet is converted to DMSeP-aldehyde, further converted to DMSeP by transamination by the chloroplast enzyme β-aldehyde dehydrogenase (Terry et al. 2000). DMSP is degraded by DMSP lyase to produce dimethylsulfide (DMS) (Diaz et al. 1992; Ledyard et al. 1993; Van Boekel and Stefels 1993; De Souza and Yoch 1995; Yoch 2002). DMSeP is postulated to be cleaved by DMSP lyase to DMSe. DMSeP-supplied plants volatilized Se at a rate of 113 times higher than that measured from plants supplied with selenate, 38 times higher than from selenite, and 6 times higher than from SeMet, suggesting DMSeP is the most likely precursor of DMSe (de Souza et al. 2000). Since MeSeMet and DMSeP are only found in Se non-hyperaccumulators, they are the precursors of DMSe volatilized by these plants. In contrast, DMDSe could likely be derived from MeSeCys selenoxide, an intermediate metabolite of MeSeCys, mainly found in Se hyperaccumulators (Terry et al. 2000). Se hyperaccumulators can accumulate large amounts of MeSeCys, γ-glutamyl-methyl-SeCys, or selenocystathionine. Therefore, DMDSe is mainly produced from MeSeCys in Se hyperaccumulators (Fig. 2).

## Rate-Limiting Enzymes for Se Volatilization

After selenate is taken up by roots via the sulfate transporters, it is transported to the leaves and enters chloroplasts, where it is metabolized by the enzymes of sulfate assimilation (Terry et al. 2000). Selenate is first reduced to adenosine 5-phosphoselenate (APSe) by ATP sulfurylase (EC: 2.7.7.4) and then further reduced nonenzymatically to GSH-conjugated selenite (GS-selenite). The GS-selenite is reduced to selenodiglutathione (GS-Se-SG) by GSH, and GS-Se-SG is further reduced to selenol (GS-SeH) by NADPH, and subsequently to GSH-conjugated selenide (GS-Se^−^) by GSH reductase (Ng and Anderson 1979). APSe could also be converted to selenite with GSH by APS reductase (EC: 1.8.99.2), and then to selenide by sulfite reductase (EC: 1.8.7.1) (Bick and Leustek 1998). SeCys is synthesized from GS-Se^−^ and O-acetylserine catalyzed by Cys synthase (Ng and Anderson 1978, 1979). In the cytoplasm, due to the presence of GSH, O-acetylserine, NADPH, and cysteine synthase, selenite may also be further converted to SeCys by Cys synthase after being reduced to GS-Se^−^ by GSH and NADPH in the cytoplasm (Nakamura et al. 1999; Foyer et al. 2001; Chai et al. 2006; Hider and Kong 2011; Li et al. 2022). SeMet is synthesized from SeCys via SeCystathionine and SeHomoCys catalyzed by cystathionine-γ-synthase, cystathionine-β-lyase, and Met synthase (Dawson and Anderson 1988). SeMet is methylated to MeSeMet and further cleaved to DMSe by S-methyl-Met hydrolase. DMSeP is most likely the precursor of DMSe, which is cleaved to DMSe by DMSP lyase (de Souza et al. 2000) (Fig. 2).

The conversion from selenate to DMSe involves at least three rate-limiting steps. The reduction of selenate is the first rate-limiting step. ATP sulfurylase catalyzes the reduction of selenate as well as sulfate in plants (Shaw and Anderson 1972; Dilworth and Bandurski 1977; Burnell 1981). The overexpression of the ATP sulfurylase gene could promote selenate reduction and accumulate more organic Se (Pilon-Smits et al. 1999). SeMet synthesis is the second rate-limiting step of selenate reduction. Se volatilization rate from the SeCys-supplied plants was almost fivefold lower than those from SeMet-supplied plants, suggesting that it is a rate-limiting step to synthesize SeMet, and Met synthase is a rate-limiting enzyme for Se volatilization. The conversion of SeMet to DMSeP is the third rate-limiting step. SeMet is first methylated by Met methyltransferase to MeSeMet, then cleaved by DMSeP lyase to DMSeP, and ultimately cleaved to DMSe by S-methyl-Met hydrolase. The rate of Se volatilization from DMSeP-supplied plants was fivefold higher than that from SeMet-supplied plants, even though the roots of plants supplied with SeMet accumulated eightfold more Se than DMSeP-supplied plants, suggesting that the conversion of SeMet to DMSeP is also a rate-limiting step and DMSeP lyase is a rate-limiting enzyme (Fig. 2). The overexpression of rate-limiting enzymes can accelerate Se volatilization (Pilon-Smits et al. 1999). Therefore, inhibiting the activity of rate-limiting enzymes may inhibit Se volatilization to a large extent.

## The Speciations of Se Compounds in Plants

Plants mainly take up selenate, selenite, and a small amount of SeMet from the soil. Selenate and selenite are mainly converted to SeCys, SeMet, and intermediate metabolites in most plants and converted to MeSeCys in a few plants such as broccoli, garlic, onion, etc. (Ávila et al. 2003; Cai et al. 1995; Lyi et al. 2005; Zhang et al. 2019). Therefore, humans can acquire Se in the form of selenate, selenite, SeCys, SeMet, MeSeCys, and intermediate metabolites from plant foods. The speciation of Se compounds has obvious different effects on preventing cancers with relative efficacy ranging from high to low as MeSeCys > selenite > SeCys > dimethylselenoxide (Ip et al. 1991). High-Se garlic has obvious anticancer efficacies. The function of high-Se garlic to prevent cancer primarily depends on the effect of Se (Ip and Lisk 1995). High-Se garlic contains Se in the form of MeSeCys (Cai et al. 1995). Feeding mice a carcinogen containing Se-enriched garlic results in a reduction in the incidence of mammary tumors in mice. This result is associated with enhanced levels of SeCys and MeSeCys in these plants (Ip et al. 1992; Cai et al. 1995). MeSeCys exhibits greater efficacy as a chemopreventive agent than several previously used Se compounds in experimental models of breast cancer (Medina et al. 2001). Rats fed supranutritional amounts of Se as broccoli exhibited greater colon cancer protection than rats fed low Se broccoli supplemented with the same amount of selenite or selenate. MeSeCys offers selective protection against organ-specific toxicity induced by clinically active agents and enhances further antitumour activity, resulting in an improved therapeutic index (Cao et al. 2014). MeSeCys is powerful in ameliorating Alzheimer’s disease-related neuropathology and cognitive deficits via modulating oxidative stress, metal homeostasis, and extracellular signal-regulated kinase activation (Xie et al. 2018). Therefore, the production of Se-enriched plant edible products mainly containing MeSeCys could improve cancer prevention. SeCys methyltransferase (SMT) in plants is responsible for the methylation of SeCys to form MeSeCys. The transgenic plants accumulated more MeSeCys by overexpressing the gene *SMT* from the Se hyperaccumulator *Astragalus bisulcatus* in Arabidopsis, Indian mustard, tobacco, and tomato than the wild type (LeDuc et al. 2004; McKenzie et al. 2009; Brummell et al. 2011). Therefore, a large amount of the cancer-preventing compound, MeSeCys, can be produced in plants by overexpressing *SMT* to meet human nutritional requirements.

## Strategies for Se Biofortification

The accumulation of Se in rice grains involves the uptake, transport, subcellular distribution, metabolism, and retanslocation of Se in rice plants. Each of these processes is crucial for grain Se accumulation. Therefore, the following strategies are proposed to improve Se accumulation in rice grains by improve Se utilization efficiency. Firstly, improving the capability of roots to take up selenite by secreting more protons. The H^+^-ATPases located in the cell membrane of the root cells are responsible for the secretion of protons. Regulating the H^+^-ATPases activity to secrete more protons can reduce the pH of the apoplastic space and the rhizosphere soil, resulting in more Se rapidly entering the root cells in the form of H_2_SeO_3_ through aquaporins. Secondly, the accumulation of Se in grains mainly involves phosphate transporters, amino acid transporters, and peptide transporters. Mining key transporters for Se accumulation and increasing the expression of these transporter genes are effective strategies to increase Se concentration in rice grains. Thirdly, regulating the distribution of Se in organelles is expected to improve the utilization efficiency of Se. When rice roots take up selenite, large amounts of Se enter the vacuole and are stored. Only when this part of Se is transported from the vacuole to the cytoplasm and then into the plastid can it be converted into organic Se and transported to the shoots. Therefore, mining the transporters responsible for Se influx and efflux located in the tonoplast and modulating their gene expression can enhance the influx of Se from the vacuole to the cytoplasm and increase the transport of more Se into the plastid. Fourthly, inhibition of Se volatilization can largely increase the Se concentration in rice grains. Se volatilization results in a large amount of Se loss from plants. Several key enzymes such as ATP sulfurylase, Met synthase, DMSeP lyase, Met methyltransferase, and S-methylMet hydrolase control the conversion from selenate to DMSe. Without interfering with normal sulfur metabolism, inhibition of certain enzyme activities is beneficial to reducing the formation of DMSe and increasing the Se concentration in rice grains. Fifthly, Se is mainly present in proteins in leaves as SeMet. Different proteases coordinate their actions to regulate protein degradation jointly during leaf senescence. It is crucial to identify the key proteases that degrade proteins to release more SeMet. Finally, the speciation of Se in plants should be optimized to make MeSeCys the main Se form in rice grains. Se exists in rice plants in the form of selenate, selenite, SeCys, SeMet, MeSeCys, and intermediate metabolites, of which MeSeCys is the most potent anticancer form. Therefore, the synthesis of MeSeCys in rice plants can be greatly enhanced by overexpressing the gene encoding SeCys methyltransferase or by gene editing its promoters by CRISPR/Cas9 to increase gene expression, thereby predominantly accumulating MeSeCys in rice grains.

In summary, enhanced Se accumulation in rice grains can be achieved by improving the efficiency of Se uptake, transport, distribution, and reutilization, and by inhibiting Se volatilization. In addition, we can also optimize the Se species that are more beneficial for human health, providing new insights and directions for the future biofortification of Se in rice.

## Abbreviations

AAP: Amino acid transporter
APSe: Adenosine 5-phosphoselenate
Cys: Cysteine
DMDSe: Dimethyldiselenide
DMSe: Dimethylselenide
DMSeP: Dimethylselenoniopropionate
DMSP: Dimethylsulfoniopropionate
GS-selenite: GSH-conjugated selenite
GS-Se-SG: Selenodiglutathione
Met: Methionine
MFS: Major facilitator superfamily
NIP: Nodulin 26-like intrinsic membrane protein
PT2: PHT1;2
PT8: PHT1;8
PTR: Peptide transporter
RuBisCO: Ribulose-1,5-bisphosphate carboxylase
Se: Selenium
SeCys: Selenocysteine
SeMet: Selenomethionine
SMT: SeCys methyltransferase
SPX: SYG1/Pho81/XPR1
VPT: Vacuolar phosphate transporter
